# A Cross-Sectional Study of Age-Related Changes in Oral Function in Healthy Japanese Individuals

**DOI:** 10.3390/ijerph17041376

**Published:** 2020-02-20

**Authors:** Kiyomi Iyota, Shinsuke Mizutani, Saori Oku, Misa Asao, Toshiko Futatsuki, Ryosuke Inoue, Yuko Imai, Haruhiko Kashiwazaki

**Affiliations:** 1 Section of Geriatric Dentistry and Perioperative Medicine in Dentistry, Division of Maxillofacial Diagnostic and Surgical Sciences, Faculty of Dental Science, Kyushu University, 3-1-1 Maidashi, Higashi-ku, Fukuoka 812-8582, Japan; kibmc@sa2.gyao.ne.jp (K.I.); saori.oku@dent.kyushu-u.ac.jp (S.O.); kashi@dent.kyushu-u.ac.jp (H.K.); 2 Iyota Dental Clinic, 2-5-5 Omiya, Chuo-ku, Fukuoka 810-0013, Japan; 3 OBT Research Center, Faculty of Dental Science, Kyushu University, 3-1-1 Maidashi, Higashi-ku, Fukuoka 812-8582, Japan; 4 Geriatric Dentistry and Perioperative Medicine in Dentistry, Kyushu University Hospital, 3-1-1 Maidashi, Higashi-ku, Fukuoka 812-8582, Japan; m.asao@dent.kyushu-u.ac.jp (M.A.); ftoshiko@dent.kyushu-u.ac.jp (T.F.); rinoue@dent.kyushu-u.ac.jp (R.I.); imaiyuko@dent.kyushu-u.ac.jp (Y.I.)

**Keywords:** cross-sectional study, oral function, independent elderly, aging

## Abstract

Background: Oral function deterioration is related to a variety of factors, including aging, decline in activities of daily living, malnutrition, and cognitive decline. This cross-sectional study examined the effects of aging on oral function in healthy individuals. Methods: A retrospective study was conducted on 175 healthy, independent patients aged 40–89 years, without dementia and with ≥20 teeth, who visited a local dental clinic in Japan. Patients were compared with 92 university students aged 20–29 years. The seven criteria proposed by the Japanese Society of Gerodontology to diagnose “oral hypofunction” were observed and statistically analyzed. Results: Compared with those in the control group, the degree of tongue coating was increased in the group aged over 80 years, occlusal force was decreased in the group aged 70–79 years, tongue motor function was decreased in the groups aged 60–69 years and older, and tongue pressure was decreased in the groups aged 70–79 years and older. Conclusions: Healthy, independent individuals maintained several oral function criteria across aging, including oral mucosal wetness, occlusal force, lip motor function, masticatory function, and swallowing function. Tongue motor function and tongue pressure decreased with aging, indicating that these may be rehabilitation targets.

## 1. Introduction

It is well known that many bodily functions deteriorate with aging, including oral functions. Although many studies have examined the effects of aging on each oral function, there has been no comprehensive report. In 2016, “oral hypofunction” was proposed as the stage where dental treatment can be performed and lead to recovery before oral dysfunction occurs. The diagnostic criteria for oral hypofunction were presented by the Japanese Society of Gerodontology (JSG) [1]. This concept involves not only an evaluation of individual items, but also a comprehensive evaluation. Oral hypofunction can be diagnosed on the basis of seven criteria. Two items pertain to the oral environment (the degree of tongue coating and oral mucosal wetness), three are individual functions (occlusal force, tongue–lip motor function, and tongue pressure), and two are comprehensive functions (masticatory and swallowing functions).

Oral function deterioration is related to a variety of factors, including aging [2,3,4], a decline in activities of daily living (ADL) [5], malnutrition [6], and cognitive decline [7]. Furthermore, individuals with 19 or fewer remaining teeth showed significantly decreased occlusal force and tongue–lip motor function compared with those with 20 or more remaining teeth. Therefore, tooth loss was associated with oral hypofunction. Although many studies have reported the effects of aging on oral function, participants had varying degrees of independence, with some participants being community dwelling [8,9,10] and some receiving home healthcare or nursing care services [2,4,10]. Therefore, it is necessary to examine aging effects on oral function after eliminating confounding factors, including decline in ADL, dementia, malnutrition, and decreased number of remaining teeth.

The current study retrospectively examined the seven criteria proposed by JSG to diagnose oral hypofunction in independent individuals over 40 years of age in a dental clinic. Changes in oral function associated with aging were examined by comparison with healthy university students. The null hypothesis of this study was that oral function can be comprehensively maintained regardless of age in independent, aging individuals without dementia, who maintain a good nutritional state, and have ≥20 teeth. Oral function was evaluated as the changes associated with aging for each investigated criteria and the comprehensive effects of aging.

## 2. Materials and Methods

### 2.1. Participants and Study Design

The participants were 175 patients who visited a dental clinic in Japan from September 2018 to June 2019 and 92 healthy university students in their 20s. For patients, the oral function examination was performed when the patient complained of spilling over the mouth, when oral function deterioration was suspected, such as choking during dental treatment, or when the patient requested. It was performed by two well-trained dental hygienists with ≥15 years of experience under the supervision of a dentist after a series of dental treatments. The results were retrospectively extracted from the dental record. The university students included those who underwent oral function examination previously. It had five inspectors, one of whom was the supervisor ensuring that all examinations were performed properly. All participants had ≥20 teeth and were independent (Barthel Index [11] = 100). Those with morphological abnormalities, dementia, or malnutrition (Mini Nutritional Assessment-Short Score [12] ≤ 7) were excluded. The patients’ age and gender are detailed in Table 1.

The design of this study was approved by the Kyushu University Institutional Review Board for Clinical Research (Approval number 2019-277). For the participants, the purpose of this study and the method of requesting exclusion from the study were posted in the university and the clinic. A sufficient opt-out period was set up. The analysis was performed using anonymized data so that individuals could not be identified.

### 2.2. Investigated Criteria

Interviews investigated ADL, nutrition status, comorbidities, and medications use. The number of remaining teeth, excluding tooth stumps and teeth with increased mobility (Miller’s classification 3 [13]), and use of dentures was measured. These investigations were performed on the same day as the oral function examination. The oral function examination was conducted and oral hypofunction was assessed on the basis of the seven criteria proposed by JSG, as discussed below.

#### 2.2.1. Degree of Tongue Coating

The degree of tongue coating was visually assessed using the Tongue Coating Index (TCI) [14]. The tongue surface was divided into nine parts, and each was assessed on a 0- to 2-point scale for degree of tongue coating, with 0 indicating the least amount. TCI % was quantified as the total score for each participant divided by the maximum score of 18.

#### 2.2.2. Oral Mucosal Wetness

An oral moisture checker (Mucus, Life Co., Ltd., Saitama, Japan) was used to measure mucosal wetness in the central area of the tongue dorsum. The tongue was pressed with a force of 200 g for 2 s for three trials. The median value was used for assessment.

#### 2.2.3. Occlusal Force

The occlusal force of the whole dentition was measured over 3 s of clenching in the intercuspal position using pressure indicating film (Dental Prescale II, GC Corporation, Tokyo, Japan) and analyzing software (Bite Force Analyzer, GC Corporation, Tokyo, Japan). For denture wearers, the measurement was performed with the dentures in place.

#### 2.2.4. Tongue–Lip Motor Function

Comprehensive motor speed and dexterity measurements for the tongue and lips were taken as oral diadochokinesis (OD). Participants were instructed to produce the syllables /pa/, /ta/, and /ka/ repeatedly for 5 s each. The number of syllables produced per second was determined using an automatic counter (Kenkokun Handy, Takei Scientific Instruments Co. Ltd., Niigata, Japan). The sounds /pa/, /ta/, and /ka/ were used to evaluate the motor function of the lip, anterior region of the tongue, and posterior region of the tongue, respectively.

#### 2.2.5. Tongue Pressure

Maximum tongue pressure was measured using a JMS tongue pressure measuring instrument (TPM-01, JMS Co. Ltd., Hiroshima, Japan). The instrument determined the maximum tongue pressure as the pressure exerted when the participants compressed a balloon attached to the tongue pressure probe onto the anterior palate for a few seconds using maximum voluntary tongue force. Measurements were taken three times, and the mean value was used for assessment. Participants who regularly wore dentures wore them during measurement.

#### 2.2.6. Masticatory Function

Glucose concentration obtained from chewed gummy jelly was measured to assess masticatory function. After chewing 2 g of gummy jelly for 20 s, participants were asked to hold 10 mL of distilled water in their mouth for a moment and to spit into a cup with a filter. The amount of eluted glucose was measured using a masticatory ability testing system (Gluco Sensor GS-II, GC Corporation, Tokyo, Japan) [15].

#### 2.2.7. Swallowing Function

Swallowing function was assessed using the self-administered “*Seirei* dysphagia screening questionnaire” [16] for swallowing screening. The questionnaire gathers pneumonia history; nutritional status; oral, pharyngeal, and esophageal swallowing phases; and the glottal defense mechanism. It is widely used to screen for eating and dysphagia.

### 2.3. Statistical Analysis

Participants were divided into six age groups: 20–29 (control), 40–49, 50–59, 60–69, 70–79, or over 80 years old.

#### 2.3.1. Gender Differences

Gender differences for each oral function criteria results within each age group were analyzed using multiple comparisons after the Kruskal–Wallis test. No gender differences were observed in any age group; therefore, male and female data were collapsed for the following analyses.

#### 2.3.2. Analysis of Oral Function

The measured values for each oral function criteria results in each age group were compared with those in the control group using chi-square tests or multiple comparisons after the Kruskal–Wallis test. Differences due to the presence or absence of dentures were compared using the same analysis method.

#### 2.3.3. Bivariate Correlation Analyses of the Criteria

Correlations between the criteria in participants who were 40 years old or older were assessed using bivariate analysis, calculating with Spearman’s rank correlation coefficient.

Values of *p* < 0.05 were considered statistically significant. The data were analyzed using SPSS version 26.0 (IBM, IBM Corporation, Tokyo, Japan).

## 3. Results

### 3.1. Participants’ Characteristics

Table 1 shows the sample size, gender, age, and the number of remaining teeth of the participants for each age group. Overall, there were 267 participants (117 men and 150 women) with an average age of 50.5 ± 22.1 (mean ± SD) years.

### 3.2. Oral Function Criteria Results

**Figure 1:**
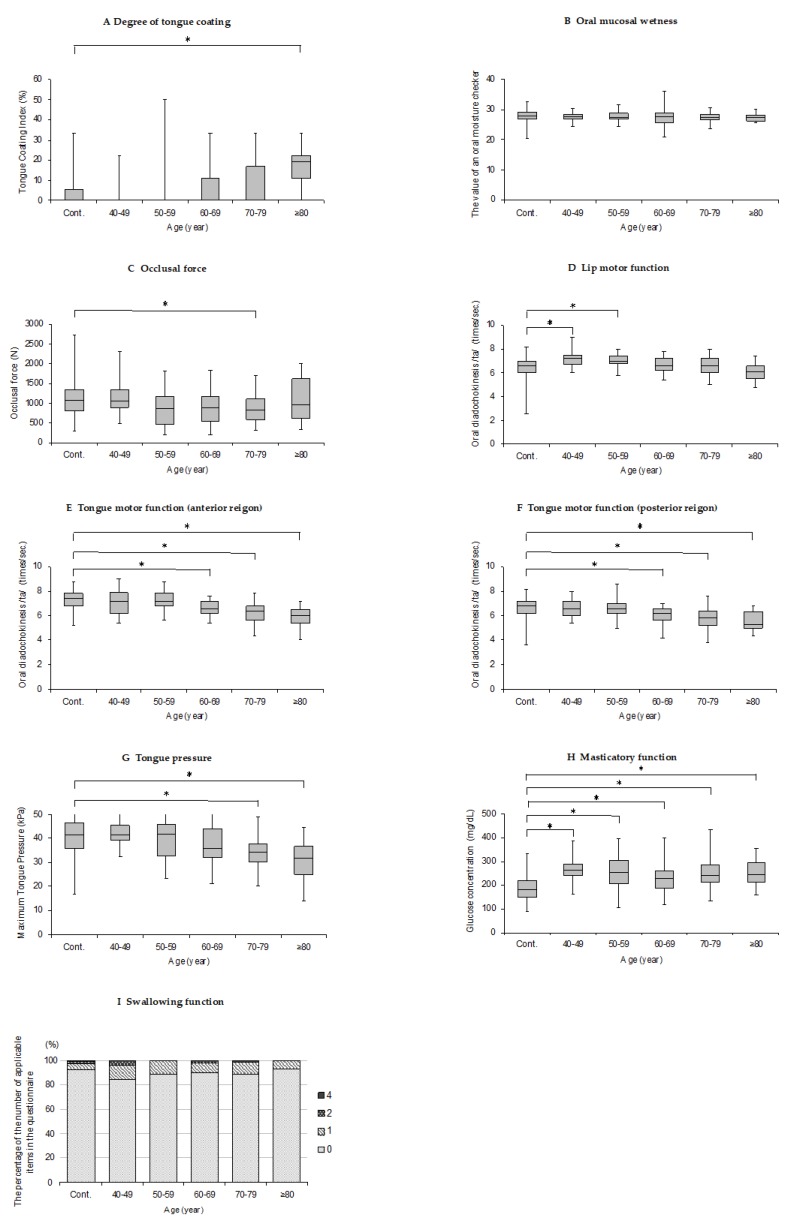
Oral function criteria results. A–H: The box plots show the value of each investigated criteria for each group. The horizontal line in the boxes is the median; the bottom of the box is the first quartile (25%), the top of the box is the third quartile (75%), and both ends of the mustache are the maximum and minimum values. I: The percentage of the number of applicable items in the questionnaire for each age group is shown. Asterisk (*) indicates *p*-value < 0.05.

The oral function criteria results are shown in Figure 1 and detailed in the following sections. No significant difference was found between the presence or absence of dentures in all the results (*p* > 0.05).

#### 3.2.1. Degree of Tongue Coating

The over 80 year-old group showed significantly higher scores compared with the control group in TCI score (*p* < 0.05; Figure 1A).

#### 3.2.2. Oral Mucosal Wetness

The age groups showed no significant differences in medial mucosal wetness scores (*p* > 0.05; Figure 1B).

#### 3.2.3. Occlusal Force

Occlusal force was significantly decreased in the 70–79 years-old group compared with that in the control group (*p* < 0.05; Figure 1C).

#### 3.2.4. Tongue–Lip Motor Function

The /pa/ OD value was significantly higher in the 40–49 year-old group and the 50–59 year-old group compared with that in the control group (*p* < 0.05; Figure 1D). The /ta/ OD value was significantly decreased in the 60–69 year-old group and older compared with that in the control group (*p* < 0.05; Figure 1E). The /ka/ OD value was significantly decreased in the 60–69 year-old group and older compared with that in the control group (*p* < 0.05; Figure 1F).

#### 3.2.5. Tongue Pressure

Tongue pressure was significantly decreased in the 70–79 year-old group and over 80 year-old group compared with that in the control group (*p* < 0.05; Figure 1G).

#### 3.2.6. Masticatory Function

The glucose concentration value was significantly higher in all other age groups compared with that in the control group (*p* < 0.05; Figure 1H).

#### 3.2.7. Swallowing Function

The age groups showed no significant differences in the response results of *Seirei* dysphagia screening questionnaire (*p* > 0.05; Figure 1I).

### 3.3. Bivariate Correlation Analyses of the Criteria

Table 2 shows a correlation analysis for age and the criteria. Age was related to OD values for /pa/, /ta/, and /ka/ and tongue pressure (r’s = −0.423, −0.480, −0.426, −0.485, respectively, *p* < 0.05). There were strong correlations between the OD value for /pa/ and /ta/ (r = 0.776, *p* < 0.05), the OD value for /pa/ and /ka/ (r = 0.765, *p* < 0.05), and the OD value for /ta/ and /ka/ (r = 0.836, *p* < 0.05).

## 4. Discussion

The current study retrospectively examined criteria related to oral function in independent patients who visited a dental clinic compared with healthy university students.

### 4.1. Degree of Tongue Coating

Ralph et al. reported that elderly individuals were more likely to exhibit a coated tongue than young adults for various reasons, including changes in dietary habits, inability to physically cope with oral hygiene techniques, a decrease in the salivary flow, and changes in the nature of the saliva [17]. However, this study found no significant differences between each group under the 70–79 year-old group nor the control group. Because there were no significant age-related differences in oral mucosa wetness in this study, it can be hypothesized that there were no changes in salivary flow or the nature of the saliva. Another factor might be that the current examinations were performed immediately following dental treatment. Yaegaki et al. reported that the amount of tongue coating in patients with periodontal disease was four times greater than that in the control group [18]. However, significantly higher TCI scores were only observed in the over 80 year-old group. The current results suggest that appropriate dental treatment might have led to a decrease in the TCI score. Kikutani et al. reported that there was a correlation between the degree of tongue coating and a reduction in tongue motor function [19]. Therefore, the current results suggest a decline in tongue motor function in the over 80 year-old group.

### 4.2. Mucosal Wetness

Although many studies have shown that aging is associated with changes in mucosal wetness, the results have been equivocal. Some have reported decreased salivary secretion with aging [20,21], whereas others have reported no effects [22,23]. Factors other than aging that contribute to dry mouth include medication use and disease comorbidity. The current study found no significant differences in oral mucosal wetness when comparing those with and without medications and comorbidities that cause dry mouth (27.7 ± 4.1, 27.6 ± 1.7, respectively). There are various assessments for dry mouth, but the degree of oral mucosal wetness was used in the current study. Scully et al. reported that medications such as antihypertensive or anxiolytic cause hyposalivation [24]. If salivary flow volume was used as an assessment, effect of comorbidities and/or use of medications might have been observed.

### 4.3. Occlusal Force

Baum et al. reported that masticatory muscle strength decreases with age [25], and some reports have indicated that there may be gender differences in these decreases. Bakke et al. concluded that occlusal force decreases with age after the age of 25 years in women and after 45 years in men [26]. Iinuma et al. reported that maximum occlusal force was significantly associated with age in men [27]. However, Shinogaya et al. reported no differences in total occlusal force distribution between groups aged 20–29 and 53–62 years with at least 28 teeth [28]. At the older end of the age spectrum, Motegi et al. reported no significant differences in bite force between the over 80 year-old with 20 or more teeth and the 60–69 years-old groups [29]. Helkimo et al. reported that the number of remaining teeth related closely with occlusal force [30]. In other words, the greater the number of remaining teeth, the greater the bite force. Although the current study found that occlusal force was significantly lower in the group aged 70–79 years compared with that in controls, it was not significantly lower in the group aged over 80 years or significantly related to age in the correlation analysis. One factor for this is that all participants had ≥20 teeth. Together, these results suggest that occlusal force might be affected by aging, but this association is not strong.

### 4.4. Tongue–Lip Motor Function

The current study revealed that the lip function, as evaluated by /pa/ OD values, was lower in the control group compared with those in the 40–49 years-old group and the 50–59 years-old group. Noro et al. reported that labial closure strength of the groups aged 30–39 and 40–49 years was higher than the groups aged 20–29 years. They hypothesized that this was due to differences in dietary habits during the growth or developmental stage; therefore, it was necessary to further examine this metric in individuals aged 20–29 years and as they aged into their 30s [31]. Similar results were obtained in the current study performed 20 years after the aforementioned report. This suggests that lip motor function might improve as the participants age into their 40s.

Conversely, tongue motor function, as evaluated by OD values of /ta/ and /ka/, significantly declined in each group after 60 years of age, which was earlier than the other criteria. Although these findings were consistent with Hara et al. [32], there was no significant stepwise decline in function after 60 years of age. Kikutani et al. also reported no significant age-related decline in /ta/ and /ka/ syllable pronunciation in individuals aged 65–88 years in the presence of posterior occlusal support [33]. These results suggest that tongue motor function begins to decline at an earlier age than the other criteria but at a slower rate.

### 4.5. Tongue Pressure

Many reports have indicated that tongue pressure decreases with aging [3,10,34], and the current study supports this finding. Maeda et al. reported that tongue pressure in the Japanese population was positively correlated with upper limb muscle mass [4]. Previous studies on age-related decreases in upper limb muscle mass differ by country. In Canadian participants, the upper limb muscle mass decreased linearly after 45 years of age in both men and women [35], whereas in American participants, this decrease began as early as in the 20s in men and women [36]. However, Japanese participants showed upper limb muscle mass decreases after their 40s in men and gradual decreases after their 30s in women [37]. Similarly, the current study found a significant correlation between age and tongue pressure in the Japanese population, which may relate to changes in upper limb muscle mass in this population.

### 4.6. Masticatory Function

Notably, masticatory function was the only oral function criteria that was significantly lower in the control group compared with that in all the other age groups. The reason for this in the current study is unclear. Kikutani et al. reported that the significant predictor of masticatory performance varies by the presence of posterior occlusal support. The predictor was the number of natural teeth in a group with posterior occlusal support, but tongue pressure in a group without support [33]. However, the number of natural teeth and tongue pressure tended to decrease with aging in the current study. In addition, /pa/ OD value was greater in the groups aged 40–49 and 50–59 years compared with that in the control group. Yamada et al. reported that shearing ability, which is required for mastication of items with consistency like gummy jelly, was not significantly correlated with /pa/ pronunciation [38]. Ikeda et al. reported that occlusal morphology flattens over aging, thereby increasing the occlusal contact area and glucose excretion in older individuals compared with those in younger individuals [39]. Therefore, further examination of factors such as occlusal contact area is required to clarify the age-related improvement in masticatory function.

### 4.7. Swallowing Function

The current study found no age-related changes in swallowing function. It has been reported that participants with dysphagia have a decline in ADL, more severe cognitive impairment, and more malnutrition than those with normal swallowing [40]. Additionally, swallowing function deterioration is present even in participants with mild dementia [7] and is associated with tooth loss [41]. Therefore, the requirements that participants be independent with good nutritional states, show no declines in cognitive function, and have ≥20 teeth for the current study may have resulted in no significant age-related changes in swallowing function.

There have been several reports that oral function deterioration is associated with frailty and sarcopenia. Watanabe et al. reported that frail elderly individuals had significantly poorer oral function than prefrail elderly individuals and robust elderly individuals [42]. Further, participants with histories of falling showed decreased occlusal force [43], and whole-body muscle has been related to tongue pressure [10] and affects sarcopenia [2]. Thus, oral and whole-body motor functions are closely related.

The current study demonstrated that many oral function criteria can be maintained regardless of age in independent elderly individuals with a good nutritional state, without a decline in cognitive function, and with ≥20 teeth. This suggests that the maintenance of oral function may be directly related to the maintenance of ADL; this maintenance might maintain good nutrition, which might indirectly lead to the maintenance of ADL. In addition, many of the elderly in this study regularly visited the dental clinic and participated in oral examination, tooth brushing instruction, and professional mechanical tooth cleaning check-ups. Their regular management and high awareness of the oral environment might have contributed to high numbers of remaining teeth and oral function maintenance. However, tongue motor function and tongue pressure still decreased with aging. Therefore, early rehabilitation to maintain these functions might prevent deterioration.

This study has some limitations. First, recruiting from a local clinic in Japan may have led to a bias in participants. In addition, because all participants were Japanese, the ability to extrapolate these findings to other ethnic groups may be limited. Second, it was a cross-sectional study that did not follow participants through the aging process. Elderly today might have better oral health than those 10 years ago. Therefore, no conclusions can be made regarding causal relationships between oral function deterioration and aging. In the future, we would like to clarify these points by conducting a cohort study with the same participants.

## 5. Conclusions

Independent elderly individuals with good nutritional states, without dementia, and with ≥20 teeth maintained several oral function criteria across the aging process, including oral mucosal wetness, occlusal force, lip motor function, masticatory function, and swallowing function. However, decreases in tongue motor function and tongue pressure were significantly, moderately correlated with age. Therefore, rehabilitation to maintain these criteria might prevent age-related oral function deterioration.

## Figures and Tables

**Table 1:** Participants’ characteristics.

Age Group	Cont.	40–49	50–59	60–69	70–79	≥80
n (men/women)	92 (52/40)	25 (10/15)	35 (9/26)	39 (18/21)	63 (21/42)	13 (7/6)
Age (year)	23.4 ± 2.1	44.2 ± 3.0	53.6 ± 2.8	65.4 ± 3.2	74.8 ± 2.5	83.7 ± 3.0
Number of remaining teeth	28.3 ± 1.8	27.6 ± 2.5	26.8 ± 2.0	25.6 ± 2.8	26.3 ± 2.4	25.1 ± 3.8
Percentage of denture users (%)	0	4.0	0	15.4	17.5	23.1

Data are shown as mean ± SD.

**Table 2:** Bivariate correlation analyses of the criteria.

	Age	TCI	Mucosal Wetness	Occlusal Force	OD/pa/	OD /ta/	OD/ka/	Tongue Pressure	Masticatory Function	Swallowing Function
Age	1	0.335 *	−0.089	−0.054	−0.423 *	−0.480 *	−0.426 *	−0.485 *	−0.089	−0.019
TCI		1	−0.078	0.078	−0.106	−0.127	−0.149	−0.218 *	0.090	0.063
Mucosal wetness			1	−0.025	0.079	0.078	0.032	0.003	−0.135	0.082
Occlusal force				1	−0.001	0.063	0.023	0.231 *	0.314 *	0.088
OD /pa/					1	0.776 *	0.765 *	0.332 *	0.160 *	−0.110
OD /ta/						1	0.836 *	0.343 *	0.182 *	−0.091
OD /ka/							1	0.294 *	0.166 *	−0.096
Tongue pressure								1	0.214 *	0.034
Masticatory function									1	−0.021
Swallowing function										1

TCI: Tongue Coating Index; OD: oral diadochokinesis; Asterisk (*) indicates *p*-value < 0.05.
